# Evaluating the Performance of the TOP100 Tool in Detecting Key Small Bowel Findings at Capsule Endoscopy

**DOI:** 10.3390/diagnostics16132005

**Published:** 2026-06-27

**Authors:** Aishwarya Devanand, Cristina Caranfil, Michaela Moore, Nicoletta Nandi, Foong Way David Tai, Melissa Hale, Christian Bentley, Victoria Thurston, Ailish Healy, Mark E. McAlindon, Reena Sidhu

**Affiliations:** 1 Academic Unit of Gastroenterology, Sheffield Teaching Hospitals NHS Foundation Trust, Sheffield S10 2JF, UK; 2 Division of Clinical Medicine, School of Medicine and Population Health, University of Sheffield, Sheffield S10 2RX, UK; 3 Department of Surgery, Oncology and Gastroenterology, University of Padova, 35124 Padova, Italy

**Keywords:** small bowel, capsule endoscopy, artificial intelligence, TOP100

## Abstract

**Background/Objectives**: Capsule endoscopy (CE) is a non-invasive tool for evaluating small bowel pathology, but prolonged reading times can lead to reader fatigue. Artificial intelligence-integrated reading software has shown promise to overcome this. This study assesses the diagnostic performance of TOP100 against standard human reading (SR) across different clinical indications. **Methods**: A retrospective single-centre cohort study was conducted at a tertiary referral centre, including patients who underwent PillCam™SB3 CE between January 2023 to August 2025. Initial reading was performed by expert CE readers (>1000 CE experience) considered as SR. Second blinded reading was performed with TOP100. CE findings from SR were compared with those from TOP100. **Results**: A total of 1382 CE were identified, of which 1374 had complete examinations and TOP100 data for analysis. The most common indications were inflammatory bowel disease (IBD) (51.5%) and iron-deficiency anaemia (IDA) (25.0%). Diagnostic yield was significantly higher with SR than TOP100 (36.0% vs. 27.5%, *p* < 0.001). TOP100 demonstrated moderate sensitivity but high specificity; 64.0% and 99.0% for active bleeding, 58.2% and 97.5% for angiodysplasia. In overt bleeding and IDA, sensitivity, and specificity for P2 lesions were 61.0% and 94.3%, respectively. Overall sensitivity and specificity were 55.3% and 95.9% for ulcers, 43.1% and 89.0% for erosions. In IBD, ulcer detection was similar, while TOP100 sensitivity for erosions improved by 9.4%. **Conclusions**: SR remains the reference standard for reading and reporting capsule endoscopies. However, TOP100 may be a useful adjunct to support interpretation and case prioritisation, especially in high-volume centres.

## 1. Introduction

Capsule endoscopy (CE) is a non-invasive diagnostic tool that enables direct endoluminal visualisation of the small bowel (SB) mucosa and its use is well established in routine gastroenterological practice [1]. It plays a pivotal role in the diagnosis of suspected SB bleeding (SSBB), SB Crohn’s disease (CD), coeliac disease, SB tumours and polyposis syndromes. The European guidelines have established clear recommendations for its use in each of these clinical settings, reflecting the growing evidence supporting its diagnostic utility [2]. Recent evidence supports, for example, the central role of CE for assessing SB and panenteric disease activity in CD, particularly in the context of treat-to-target strategies and mucosal healing [3,4]. In overt SSBB, CE is the first line investigation recommended following negative bidirectional endoscopy. However, its diagnostic yield is time-sensitive, with evidence demonstrating that CE performed within 48 h of the bleeding episode yields a diagnostic rate approximately 20% higher than CE performed after 72 h [5]. Given the complexity and often nonspecific presentation of SB diseases, the importance of CE as a diagnostic tool should be strongly emphasised, while clear and optimised diagnostic pathways should be further defined.

Despite its clinical utility, the substantial time required to review images is one of the main limitations of CE and may also affect detection rates, even among expert readers. A standard examination generates approximately 50,000 images over an average 8 h recording period and requires between 30 min and 2 h for complete review, depending on reader experience and lesion complexity [6]. The large image volume and repetitive nature of consecutive frames require prolonged periods of sustained attention to accurately identify SB pathology. This cognitive demand contributes to reader fatigue, which has shown to adversely affect diagnostic performance, with lesion detection decreasing by up to 20% following review of a single CE study [7].

Artificial intelligence (AI), particularly computer-aided detection (CADe) systems, has been introduced into CE reading platforms to address the interpretive challenges associated with capsule review. In a large multicentre landmark study, Ding et al. [8] reported a per-patient sensitivity of 99.88% for AI-assisted detection of SB pathology, supporting its diagnostic reliability in clinical practice. This is reinforced by pooled comparative analyses demonstrating higher sensitivity with AI-supported interpretation than standard human read (SR), 92% and 86% respectively, alongside improved negative predictive values [9]. In addition to enhancing lesion detection, AI has been shown to improve reading efficiency through automated frame prioritisation. Recordings comprising a mean of 22,654 images have been condensed to approximately 578 abnormal frames per examination following prioritisation [8]. By substantially decreasing the number of images requiring review, this approach has the potential to reduce interpretation time and mitigate reader fatigue while maintaining diagnostic accuracy. Collectively, these findings support AI-assisted CE review as a reliable and efficient adjunct to conventional CE interpretation, further reinforced by the 2025 ESGE Quality Improvement Initiative supporting its use alongside conventional capsule reads [10].

**Figure 1:**
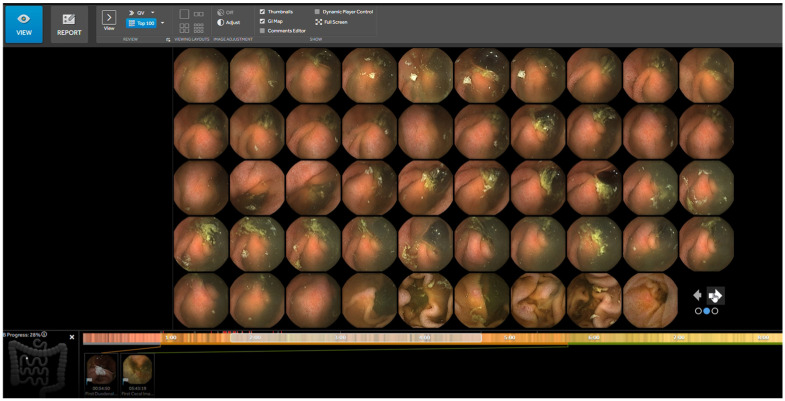
TOP100 Medtronic PillCam™ SB platform.

Within commercially available CE systems, structured image selection AI-based tools such as the TOP100 (Medtronic PillCam™ SB platform) have been developed to facilitate targeted evaluation of key frames. Following the identification of the first duodenal and caecal images, the system automatically selects 100 frames most likely to contain clinically relevant SB pathology, presenting a condensed image set for clinician review (Figure 1).

Although AI-assisted CE demonstrates high overall diagnostic performance, detection rates vary according to lesion characteristics and clinical indications. For instance, SB polyps are associated with higher miss rates than ulcers or vascular lesions, largely due to their subtle morphology and visual similarity to surrounding mucosa [11]. While this variability is well recognised, the performance of a single prioritisation-based tool, such as TOP100, across lesion types and clinical contexts has not been comprehensively evaluated, and its ability to mitigate established variation in detection remains uncertain.

The aim of this study was to assess the detection of SB lesions using the TOP100 feature, namely angioectasia, ulcers, tumours, polyps, active bleeding, erosions, bulges, strictures, mucosal atrophy, and diverticula, compared with the gold standard (SR) in a real-world clinical setting.

## 2. Materials and Methods

### 2.1. Study Design

We performed a retrospective, single-centre cohort study, including all consecutive patients who underwent SB CE using the PillCam™ (Medtronic, Dublin, Ireland) SB3 system across all clinical indications between January 2023 and August 2025 at the Royal Hallamshire Hospital, Sheffield Teaching Hospitals NHS Foundation Trust. Procedures were performed as a part of routine clinical care in accordance with European Society of Gastrointestinal Endoscopy (ESGE) guidelines. All the adult patients aged older than 16 years who attended our service for CE during the study period were included. Patients <16-year-old, inability to swallow the capsule and the absence of an associated TOP100 view required for the secondary read were our exclusion criteria.

Capsule recordings and corresponding SR reports were archived within the institutional CE database, enabling retrospective analysis. Abnormalities documented within the original SR were extracted and classified according to predefined lesion categories (angioectasia, ulcers, tumours, polyps, active bleeding, erosions, bulges, strictures, mucosal atrophy, and diverticula); bleeding potential of lesions was assessed according to Saurin classification, which classifies lesions according to their potential of clinically significant bleeding (P0: no bleeding potential, P1: uncertain bleeding potential, P2: high bleeding potential) [12]. All procedures were assessed as to whether they were diagnostic based on clinician judgement, taking into account clinical history, the presence of clinically relevant findings and their implications on subsequent patient management. Specifically, the predefined criteria for a positive diagnosis differed according to the indication. Angioectasia, ulcers, erosions and active bleeding, according to the Saurin classification, were considered diagnostically positive for overt bleeding and IDA. Atrophy was considered the characteristic feature of coeliac disease. In the context of IBD, ulcers, erosions and strictures were considered the defining pathological lesions. Finally, in patients with suspected SB lesions, polypoid lesions were considered positive diagnostic lesions. Red dots, white tipped villi, phlebectasia, and lymphangectasia were not included, as they were not considered to be clinically relevant. Each CE study subsequently underwent a secondary assessment using the TOP100 AI-assisted image selection feature integrated within the PillCam™ reading software (v9.7). This review was performed by an expert CE reader (with an experience of more than 500 CE examinations) who was blinded to the original CE report and independent of the original reporting clinicians; only the AI-selected images were reviewed. Abnormalities identified within these images were recorded according to the same predefined lesion categories, and the diagnostic value of each procedure was assessed using the same approach as SR data extraction after completion of the TOP100 read, without blinding to the clinical indication.

### 2.2. Capsule Endoscopy Procedure

**Figure 2:**
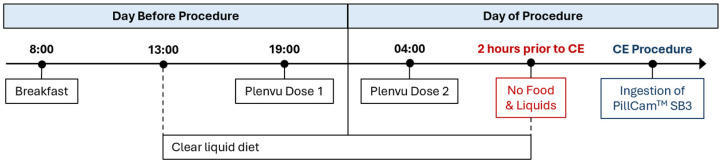
Bowel preparation protocol for CE.

All patients underwent PillCam™SB3 CE. As per protocol, patients received a polyethene glycol (PEG)-based bowel preparation (Plenvu), which was administered in two doses to improve bowel cleansing. In some cases, patients followed a clear liquid diet for 24 h prior to the examination, with intake restricted to water only from 9:00 p.m. on the evening preceding the procedure. However, this was not routinely required for all patients, but mostly used in frail and elderly patients, patients with known poor bowel preparation tolerance and in urgent cases (“hot capsules”). All oral intake was stopped two hours prior to capsule ingestion (Figure 2).

### 2.3. Data Collection

Clinical and procedural data were obtained from electronic patient records and the institutional CE database.

Clinical variables comprised patient demographics such as age and sex, prior colonoscopy or oesophagogastroduodenoscopy (OGD) within 5 years of the CE and relevant comorbidities including known or suspected inflammatory bowel disease (IBD), aortic stenosis, chronic kidney disease, atrial fibrillation, ischemic heart disease and hereditary haemorrhagic telangiectasia. Findings from previous directional endoscopic investigations were recorded where available, alongside haemoglobin and ferritin levels when measured within 3 months of the procedure.

Procedural variables included the clinical indication, study completion defined as visualisation of the caecum within the capsule recording time, adequacy of bowel preparation according to the reader and small bowel transit time. Lesion findings from both the standard reading report and the AI-assisted TOP100 review were extracted and compared.

### 2.4. Statistics

Statistical analysis was performed using Stata version 18 (StataCorp, College Station, TX, USA). The normality of continuous variables was assessed using the Shapiro–Wilk test. Normally distributed variables are reported as mean ± standard deviation, whereas non-normally distributed variables are presented as median and interquartile range (IQR). Categorical variables are summarised as frequencies and percentages.

Diagnostic yield was calculated for both the SR and TOP100 and compared between the two modalities. Diagnostic accuracy analysis of TOP100 for SB findings was performed on an examination-level basis and using the SR as the reference standard. Each finding identified was recorded as present or absent according to both the TOP100 and SR. Multiple lesions of the same type within a single examination were not counted separately. Sensitivity, specificity, positive predictive value (PPV) and negative predictive value (NPV) were calculated for each SB finding. Additional subgroup analyses were performed to evaluate performance across clinical indications using the same diagnostic accuracy measures.

Ninety-five percent confidence intervals (95% CIs) were calculated for all diagnostic accuracy measures, and a *p*-value < 0.05 was considered statistically significant.

## 3. Results

### 3.1. Study Population

A total of 1382 CE procedures were performed between January 2023 and August 2025. Of these, eight procedures were excluded: four due to the inability to swallow the capsule and four due to the absence of an associated TOP100 view required for the secondary read. The final study cohort comprised 1374 CE procedures performed in 1248 patients, of whom 115 underwent repeat examinations during the study period. The median patient age was 52 years (IQR: 33–65) and majority were female (56.6%, *n* = 778). The most common indications for CE were suspected or established IBD (51.5%) and IDA (25.0%). Less frequent indications included overt gastrointestinal bleeding (6.8%), followed by suspected polyposis or small bowel masses and investigation for complications of coeliac disease (both 5.5%). Inflammatory bowel disease was the most prevalent comorbidity, affecting 25.9% of patients, while the prevalence of all other comorbidities was less than 10% (Table 1).

Prior to CE, most patients underwent bidirectional endoscopic evaluation. Colonoscopy was performed in 75.8% of patients (*n* = 1042), while OGD was carried out in 53.7% (*n* = 738). Across both endoscopic procedures, inflammatory changes were the most commonly observed abnormalities, identified in 23.5% (*n* = 245) and 6.6% (*n* = 49) of colonoscopies and OGDs, respectively. A potential bleeding source was detected in 5.7% (*n* = 60) of colonoscopies and 5.1% (*n* = 37) of OGDs. Despite these findings, therapeutic intervention was infrequent, being undertaken in only 4.1% (*n* = 43) of colonoscopies and 3.3% (*n* = 24) of OGDs.

### 3.2. Capsule Endoscopy

The CE examination was complete in 90.8% (*n* = 1248) of the included procedures, with a median small bowel transit time of 222 min (IQR 149–279 min). Small bowel preparation was deemed adequate in 89.4% (*n* = 1229) of the CE recordings.

### 3.3. Overall Diagnostic Yield and Performance of TOP100 Per-Lesion

**Figure 3:**
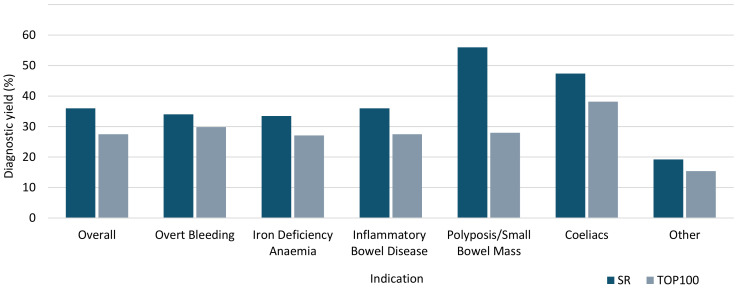
Bar chart showing the diagnostic yield of standard reading (SR) vs. TOP100 across capsule endoscopy indication.

Standard reading yielded a significantly higher overall diagnostic rate than the TOP100 review (36.0% vs. 27.5%, *p* < 0.001); see Table 2 and Figure 3. The diagnostic performance of the TOP100 varied by lesion type (Table 3). Overall, specificity was uniformly high across all lesion categories (89.0%), reaching ≥99.0% for tumours, bulges, strictures, active bleeding and diverticula, whereas sensitivity showed greater variability (8.8–64.0%). For the frequencies of each lesion according to the different indication see Tables S4 and S5.

The highest sensitivity was achieved in the detection of active bleeding (64.0%, 95% CI: 49.2–77.1%), with a corresponding PPV and NPV of 71.1% and 98.6%. Angioectasias and ulcers also demonstrated relatively high sensitivity (58.2% [95% CI: 49.9–66.1%] and 55.3% [95% CI: 49.8.6–60.6%]), with specificity of 97.5% and 95.9% and PPVs of 74.2% and 81.8%, respectively. However, the NPV for ulcers (86.6%, 95% CI: 84.5–88.5%) was lower than that for angioectasias (94.9%, 95% CI: 93.5–96.0%).

In contrast, sensitivity for the remaining lesion types were consistently lower (<50.0%). Erosions were detected with a sensitivity of 43.1% (95% CI: 38.1–48.2%) and a specificity of 89.0% (95% CI: 86.9–90.9%) and were associated with the lowest NPV among all lesion categories (80.2%, 95% CI: 77.7–82.5%). Strictures and atrophy showed modest sensitivity of 38.6% (95% CI: 24.4–54.5%) and 36.3% (95% CI: 27.8–45.4%), but maintained high specificity (99.5% and 98.1%), with NPVs of 98.0% and 93.9%, respectively. Tumours (20.2%, 95% CI: 12.3–30.4%), diverticula (13.0%, 95% CI: 2.8–33.6%) and bulges (8.8%, 95% CI: 2.9–19.3%) showed the lowest sensitivity, despite very high specificity (99.4–99.9%), with PPVs of 68.0%, 75.0%, and 50.0%, respectively and NPVs ranging from 95.0% to 98.5%.

### 3.4. Diagnostic Performance of TOP100 Per-Indication

#### 3.4.1. Inflammatory Bowel Disease

In the IBD subgroup, the overall diagnostic yield was significantly lower with TOP100 (36.0% vs. 27.5%, *p* < 0.001). Sensitivity across lesion types in the IBD cohort reached 59.1% for ulcers, 52.5% for erosions and 43.5% for strictures. Specificity remained high throughout all IBD lesion types, exceeding 85%, with the lowest observed for erosions. The PPV increased for ulcers and erosions but was lower for stricture compared with the overall cohort. The NPV remained high, ranging from 81.1% to 99.6% (Table S1).

#### 3.4.2. Overt Bleeding and Iron Deficiency Anaemia

In patients investigated for overt bleeding and IDA, overall TOP100 in comparison to SR achieved a diagnostic yield of 29.8% vs. 34.0% (*p* = 0.424) and 27.1% vs. 33.5% (*p* = 0.012), respectively. The TOP100 detection of active bleeding showed a good sensitivity (72.7% in overt bleeding and 61.5% in IDA), and high specificity (97.6% and 98.7%, respectively), with identical PPV (80.0%) and comparable NPV across both groups (96.4% and 96.9%).

Performance for angioectasias was characterised by moderate sensitivity (51.9–67.5%) and consistently high specificity (>95%). PPV exceeded that of the overall cohort (82.4% in overt bleeding and 87.1% in IDA), whereas NPV was lower (83.1% and 90.7% vs. 94.9% overall).

In contrast, detection of erosions was less sensitive (29.4% in overt bleeding and 29.2% in IDA, vs. 43.1% overall), and associated with lower PPV (45.5% and 58.3% vs. 60.2% overall), although specificity and NPV remained high (>90% and >80%, respectively). When considering Saurin classification, P2 lesions yielded a sensitivity of 61.0%, specificity of 94.3%, PPV of 89.3%, and NPV of 75.8% (Table S2).

#### 3.4.3. Coeliac Disease

In patients undergoing CE for coeliac disease, the overall diagnostic yield of TOP100 compared to SR was 38.2% vs. 47.4% (*p* = 0.230), and detection of atrophy showed higher sensitivity among the coeliac disease subgroup compared with the overall cohort (59.3% vs. 36.3%), and a higher PPV (91.4% vs. 65.2%), with a slight reduction in specificity (86.4%) and lower NPV (46.3%). Conversely, sensitivity for ulcer detection was reduced in the coeliac cohort (37.5% vs. 55.3%), although specificity, PPV and NPV were higher, with both specificity and positive predictive value reaching 100.0% (Table S1).

#### 3.4.4. Polyposis Syndromes and Small Bowel Masses

Within the subgroup investigated for polyposis or suspected small bowel masses, the TOP100 reading showed a significantly lower diagnostic yield 28.0% vs. 56.0% (*p* < 0.001). However, it achieved improved sensitivity for both bulges and tumours/polyps compared with the overall cohort (15.0% and 50.0%, respectively). This was accompanied by a corresponding increase in PPVs: from 50.0% to 100.0% for bulges and from 68.0% to 92.9% for tumours/polyps. Specificity remained comparable to overall estimates, while NPV was largely unchanged, with the exception of a modest reduction for tumour detection (78.7%) (Table S3).

## 4. Discussion

The present study provides a comprehensive assessment of TOP100 performance by evaluating its diagnostic value across all ESGE-recommended indications in a real-world setting. This broader approach expands the available evidence and addresses an important gap in the current literature by providing insight into the performance of TOP100 in a tertiary referral centre.

In the present study, the diagnostic yield of the TOP100 was lower at 27.5% compared to that of the SR of 36%. However, it consistently demonstrated high specificity and PPV across lesion types and clinical indications, supporting its ability to confirm pathology when detected. In patients investigated for overt bleeding, TOP100 showed good sensitivity (72.7%) and high specificity (97.6%) for active bleeding.

Within the IBD subgroup, ulcer detection was comparable between TOP100 and SR (59.1% vs. 55.3%), with slightly higher sensitivity for erosions and strictures observed with TOP100 (9.4% and 4.9%, respectively).

In coeliac disease, where TOP100 has not previously been evaluated, detection of villous atrophy was higher in the coeliac cohort compared with the overall cohort (59.3% vs. 36.3%), suggesting improved identification of diffuse mucosal changes. In suspected SB tumours and polyposis, sensitivity remained low despite high specificity, although detection was higher within this cohort than overall (tumours 50.0% vs. 20.2%, bulges 15.0% vs. 8.8%), highlighting persistent diagnostic limitations. To our knowledge, for the first time these findings provide novel insight into TOP100 performance in previously underexplored indications.

The high DY observed with SR suggests that TOP100 may miss some clinically relevant findings compared with conventional reading strategies in routine clinical practice. Furthermore, a negative TOP100 result cannot reliably exclude significant pathology, but it may be a useful adjunct to support interpretation and case prioritisation, especially in high-volume centres.

The growing body of evidence on AI-assisted CE suggests that automated image analysis may substantially improve the efficiency of video review while maintaining acceptable diagnostic performance across a range of clinical indications. Our findings are broadly consistent with those reported by Giordano et al. [13], who reported a DY of 75.7% for SR vs. 68.5% for TOP100 in overt GI bleeding.

Similarly, Freitas et al. [14] reported comparable DY for clinically significant inflammatory lesions between SR (55.7%) and TOP100 (47.0%), alongside a higher ulcer detection rate (73.6%), although some mild inflammatory activity was not detected by TOP100.

Beyond SB CE, the performance of TOP100 has also been investigated in colon capsule and panenteric capsule endoscopy. In a recent study [15], TOP100 demonstrated high sensitivity for the detection of colorectal lesions in a cohort of 188 patients with a previous incomplete colonoscopy, supporting its potential role as a triage tool to prioritise CE review in high-volume centres.

Expanding the application of AI platforms to the entire enterocolonic tract, Rosa et al. [16] reported good diagnostic performance compared with conventional capsule endoscopy reading and colonoscopy (60% vs. 42%, *p* < 0.01) in a cohort of 100 patients investigated for suspected mid–lower GI bleeding, highlighting the growing potential of AI-assisted systems in gastrointestinal endoscopy.

In addition to TOP100, the benefits associated with AI-assisted reading have been investigated across different platforms. Mushtaq et al. [17] showed that AI-assisted double-headed capsule endoscopy (MiroCam MC2000) improved diagnostic accuracy (52%) and specificity (96%), while significantly reducing reading time, supporting the use of AI as an adjunct to expert interpretation, complementing rather than replacing clinical decision-making. Likewise, Spada et al. [18] demonstrated in a prospective multicenter study that the ProScan AI platform integrated into the Navicam SB system achieved diagnostic performance comparable to conventional reading methods, while reducing mean reading time from 33.7 to 3.8 min.

The following limitations should be acknowledged in relation to this study. Its retrospective and single-centre design may limit the generalisability of the findings. In addition, TOP100 and SR were performed independently by different readers rather than by the same reader using both methods. This introduces inter-reader variability and makes direct comparison between approaches more difficult, as differences in detection may be influenced by individual reader interpretation and experience. Consequently, some of the observed differences may reflect reader-related factors rather than true differences in diagnostic performance. Per-patient analysis was not performed, as the study focused on per-lesion diagnostic yield, which limits assumptions regarding decision-making across CE indications. Moreover, a small proportion of patients underwent repeated CE examinations and, since analyses were performed at a per-examination level, repeated procedures from the same patient were still included in the analysis. However, these were only 9%, making a substantial impact on the results unlikely.

Finally, a consensus reference standard, established through independent review of all CE videos by multiple expert readers and resolution of any disagreements, would have provided a more robust comparator for evaluating AI-assisted performance.

On the other hand, this study has several strengths, including a large cohort from a tertiary referral centre, which supports the reliability and clinical relevance of the findings. The inclusion of detailed subgroup analyses across multiple indications allows for a more comprehensive assessment of TOP100 performance. All the CE readers in the study had vast experience in reading and reporting CE within a busy service.

## 5. Conclusions

TOP100 may be a useful adjunct to support interpretation and case prioritisation of CE reviews, especially in high-volume centres. Further iterative refinement of AI tools, including TOP100 and similar AI-based platforms, may therefore be necessary to achieve optimal diagnostic accuracy, with performance likely to improve across successive updates.

## Figures and Tables

**Table 1:** Demographic characteristics of the study population.

Characteristics	*N* = 1374 *N* (%)
Age (years) ^‡^	52 (33, 65)
Sex	
Female	778 (56.6)
Male	596 (43.4)
Indication for CE	
Overt bleeding	94 (6.8)
Iron deficiency anaemia	343 (25.0)
Inflammatory bowel disease	708 (51.5)
Polyposis/small bowel mass	75 (5.5)
Coeliac disease	76 (5.5)
Other	78 (5.7)
Comorbidities	
Coeliac disease	85 (6.2)
Inflammatory bowel disease	356 (25.9)
Aortic stenosis	23 (1.7)
Chronic kidney disease	106 (7.7)
Atrial fibrillation	61 (4.4)
Ischaemic heart disease	64 (4.7)
Hereditary Hemorrhagic Telangiectasia	2 (1)
Previous endoscopic investigations	
Colonoscopy	
Yes	1042 (75.8)
No	332 (24.2)
Oesophagogastroduodenoscopy	
Yes	738 (53.7)
No	636 (46.6)
Complete CE procedure	1248 (90.8)
SBTT ^‡^	222 min (149–279 min)
Small bowel preparation	
Adequate	1229 (89.4)
Inadequate	145 (10.6)

^‡^ Median (interquartile range), SBTT small bowel transit time, CE capsule endoscopy.

**Table 2:** Diagnostic yield of standard reading (SR) vs. TOP100 across capsule endoscopy indication.

	SR	TOP100	*p*-Value
Overall	36.0%	27.5%	*p* < 0.001
Overt bleeding	34.0%	29.8%	*p* = 0.424
Iron deficiency anaemia	33.5%	27.1%	*p* = 0.012
Inflammatory bowel disease	36.0%	27.5%	*p* < 0.001
Polyposis/small bowel mass	56.0%	28.0%	*p* < 0.001
Coeliacs	47.4%	38.2%	*p* = 0.230
Other	19.2%	15.4%	*p* = 0.607

**Table 3:** Overall diagnostic performance of TOP100 across lesion type.

Lesions	% (95% CI)			
	Sensitivity	Specificity	PPV	NPV
Angioectasia	58.2 (49.9–66.1)	97.5 (96.4–98.3)	74.2 (65.4–81.7)	94.9 (93.5–96.0)
Ulcer	55.3 (49.8–60.6)	95.9 (94.5–97.1)	81.8 (76.2–86.6)	86.6 (84.5–88.5)
Tumour/Polyp	20.2 (12.3–30.4)	99.4 (98.8–99.7)	68.0 (46.5–85.1)	95.0 (93.7–96.1)
Active bleeding	64.0 (49.2–77.1)	99.0 (98.3–99.5)	71.1 (55.7–83.6)	98.6 (97.9–99.2)
Erosions	43.1 (38.1–48.2)	89.0 (86.9–90.9)	60.2 (54.2–66.1)	80.2 (77.7–82.5)
Bulge	8.8 (2.9–19.3)	99.6 (99.1–99.9)	50.0 (18.7–81.3)	96.2 (95.0–97.1)
Stricture	38.6 (24.4–54.5)	99.5 (98.9–99.8)	70.8 (48.9–87.4)	98.0 (97.1–98.7)
Atrophy	36.3 (27.8–45.4)	98.1 (97.2–98.8)	65.2 (52.8–76.3)	93.9 (92.5–95.2)
Diverticula	13.0 (2.8–33.6)	99.9 (99.6–100.0)	75.0 (19.4–99.4)	98.5 (97.8–99.1)
P2 Lesions *	61.0 (51.1–70.8)	94.3 (89.7–99.0)	89.3 (83.0–95.5)	75.8 (67.1–84.4)

* diagnostic performance calculated among patients with IDA and overt bleeding.

## Data Availability

The data presented in this study are available on request from the corresponding author due to privacy and ethical restrictions.

## Supplementary Materials

The following supporting information can be downloaded at: https://www.mdpi.com/article/10.3390/diagnostics16132005/s1, Table S1: Diagnostic performance of TOP100 in IBD and coeliac disease; Table S2: Diagnostic performance of TOP100 in overt bleeding and IDA; Table S3: Diagnostic performance of TOP100 in polyposis and suspected SB mass; Table S4: Frequency of lesions identified by clinical indication across standard reading (SR); Table S5: Frequency of lesions identified by clinical indication across AI-assisted reading (TOP100).

## Abbreviations

The following abbreviations are used in this manuscript:

AI	Artificial intelligence
CE	Capsule endoscopy
DY	Diagnostic yield
IBD	Inflammatory bowel disease
IDA	Iron deficiency anaemia
NPV	Negative predictive value
OGD	Oesophagogastroduodenoscopy
PPV	Positive predictive value
SB	Small bowel
SSBB	Suspected small bowel bleeding
SR	Standard reading
