# Supplementary material for: Evaluating the Performance of the TOP100 Tool in Detecting Key Small Bowel Findings at Capsule Endoscopy
Source: Diagnostics (Basel). 2026 Jun 27;16(13):2005. doi: 10.3390/diagnostics16132005 (PMC13360472; doi:10.3390/diagnostics16132005)
Supplement: Supplementary file 1 [file diagnostics-16-02005-s001.zip › diagnostics-4343671-supplementary.pdf]

Table S1. Diagnostic performance of TOP100 in IBD and coeliac disease

Lesion	IBD				Coeliac disease			
	**Sensitivity** % (95% CI)	**Specificity** % (95% CI)	**PPV** % (95% CI)	**NPV** % (95% CI)	**Sensitivity** % (95% CI)	**Specificity** % (95% CI)	**PPV** % (95% CI)	**NPV** % (95% CI)
**Angioectasia**	42.9 (24.5-62.8)	98.4 (97.1-99.2)	52.2 (30.6-73.2)	97.7 (96.2-98.7)	71.4 (29.0-96.3)	91.3 (82.0-96.7)	45.5 (16.7-76.6)	96.9 (89.3-99.6)
**Ulcer**	59.1 (52.7-65.3)	94.1 (91.6-96.1)	84.4 (78.1-89.5)	81.1 (77.5-84.4)	37.5 (8.5-75.5)	100.0 (94.7-100.0)	100.0 (29.2-100.0)	93.2 (84.7-97.7)
**Tumour/polyp**	0 (0.0-16.1)	99.7 (99.0-100.0)	0 (0.0-84.2)	97.0 (95.5-98.1)	- -	- -	- -	- -
**Active bleeding**	50.0 (11.8-88.2)	99.3 (98.3-99.8)	37.5 (8.5-75.5)	99.6 (98.8-99.9)	100.0 (15.8-100.0)	97.3 (90.6-99.7)	50.0 (6.8-93.2)	100.0 (95.0-100.0)
**Erosions**	52.5 (46.0-58.9)	85.0 (81.4-88.1)	64.5 (57.4-71.1)	77.5 (73.6-81.0)	17.6 (3.8-43.4)	84.7 (73.0-92.8)	25.0 (5.5-57.2)	78.1 (66.0-87.5)
**Bulge**	0 (0.0-20.6)	99.9 (99.2-100.0)	0 (0.0-97.5)	97.7 (96.4-98.7)	- -	- -	- -	- -
**Stricture**	43.5 (23.2-65.5)	99.1 (98.1-99.7)	62.5 (35.4-84.8)	98.1 (96.8-99.0)	- -	- -	- -	- -
**Atrophy**	14.3 (4.8-30.3)	98.5 (97.3-99.3)	33.3 (11.8-61.6)	95.7 (93.9-97.1)	59.3 (45.0-72.4)	86.4 (65.1-97.1)	91.4 (76.9-98.2)	46.3 (30.7-62.6)
**Diverticula**	25.0 (3.2-65.1)	99.9 (99.2-100.0)	66.7 (9.4-99.2)	99.1 (98.2-99.7)	- -	- -	- -	- -

Table S2. Diagnostic performance of TOP100 in overt bleeding and IDA

Lesions	Overt bleeding				IDA			
	Sensitivity % (95% CI)	Specificity % (95% CI)	PPV % (95% CI)	NPV % (95% CI)	Sensitivity % (95% CI)	Specificity % (95% CI)	PPV % (95% CI)	NPV % (95% CI)
**Angioectasia**	51.9 (31.9-71.3)	95.5 (87.5-99.1)	82.4 (56.6-96.2)	83.1 (72.9-90.7)	67.5 (56.1-77.6)	97.0 (94.1-98.7)	87.1 (76.1-94.3)	90.7 (86.7-93.8)
**Ulcer**	36.4 (10.9-69.2)	97.6 (91.6-99.7)	66.7 (22.3-95.7)	92.0 (84.3-96.7)	52.7 (38.8-66.3)	96.9 (94.2-98.6)	76.3 (59.8-88.6)	91.5 (87.8-94.4)
**Tumour/polyp**	20.0 (0.5-71.6)	98.9 (93.9-100.0)	50.0 (1.3-98.7)	95.7 (89.2-98.8)	4.0 (0.1-20.4)	98.7 (96.8-99.7)	20.0 (0.5-71.6)	92.9 (89.6-95.4)
**Active bleeding**	72.7 (39.0-94.0)	97.6 (91.6-99.7)	80.0 (44.4-97.5)	96.4 (89.9-99.3)	61.5 (40.6-79.8)	98.7 (96.8-99.7)	80.0 (56.3-94.3)	96.9 (94.4-98.5)
**Erosions**	29.4 (10.3-56.0)	92.2 (83.8-97.1)	45.5 (16.7-76.6)	85.5 (76.1-92.3)	29.2 (19.0-41.1)	94.5 (91.0-96.9)	58.3 (40.8-74.5)	83.4 (78.7-87.4)
**Bulge**	50.0 (1.3-98.7)	98.9 (94.1-100.0)	50.0 (1.3-98.7)	98.9 (94.1-100.0)	7.1 (0.2-33.9)	99.4 (97.8-99.9)	33.3 (0.8-90.6)	96.2 (93.6-97.9)
**Stricture**	- -	- -	- -	- -	38.5 (13.9-68.4)	99.7 (98.3-100.0)	83.3 (35.9-99.6)	97.6 (95.4-99.0)
**Atrophy**	- -	- -	- -	- -	13.6 (2.9-34.9)	98.1 (96.0-99.3)	33.3 (7.5-70.1)	94.3 (91.3-96.5)
**Diverticula**	16.7 (0.4-64.1)	100.0 (95.9-100.0)	100.0 (2.5-100.0)	94.6 (87.9-98.2)	- -	- -	- -	- -

Table S3. Diagnostic performance of TOP100 in polyposis and suspected SB mass

Lesions	Polyposis and suspected SB mass			
	**Sensitivity** % (95% CI)	**Specificity** % (95% CI)	**PPV** % (95% CI)	**NPV** % (95% CI)
**Angioectasia**	16.7 (0.4-64.1)	100.0 (94.8-100.0)	100.0 (2.5-100.0)	93.2 (84.9-97.8)
**Ulcer**	28.6 (8.4-58.1)	95.1 (86.3-99.0)	57.1 (18.4-90.1)	85.3 (74.6-92.7)
**Tumour/polyp**	50.0 (29.9-70.1)	98.0 (89.1-99.9)	92.9 (66.1-99.8)	78.7 (66.3-88.1)
**Active bleeding**	33.3 (0.8-90.6)	100.0 (95.0-100.0)	100.0 (2.5-100.0)	97.3 (90.6-99.7)
**Erosions**	33.3 (13.3-59.0)	93.0 (83.0-98.1)	60.0 (26.2-87.8)	81.5 (70.0-90.1)
**Bulge**	15.0 (3.2-37.9)	100.0 (93.5-100.0)	100.0 (29.2-100.0)	76.4 (64.9-85.6)
**Stricture**	25.0 (0.6-80.6)	100.0 (94.9-100.0)	100.0 (2.5-100.0)	95.9 (88.6-99.2)
**Atrophy**	66.7 (9.4-99.2)	98.6 (92.5-100.0)	66.7 (9.4-99.2)	98.6 (92.5-100.0)
**Diverticula**	- -	- -	- -	- -

Table S4. Frequency of lesions identified by clinical indication across standard reading (SR)

Lesion	Overall *N*	SR *N (%)*				
		Overt Bleeding	IDA	IBD	Polyposis/ SB masses	Coeliac
**Angioectasia**	153	27 (17.6)	80 (52.2)	28 (18.3)	6 (3.9)	7 (4.6)
**Ulcer**	342	11 (3.4)	55 (16.0)	247 (72.2)	14 (4.1)	8 (2.3)
**Tumour/Polyp**	84	5 (5.9)	25 (29.7)	21 (25.0)	26 (30.1)	4 (4.8)
**Active Bleeding**	50	11 (22.0)	26 (52.0)	6 (12.0	3 (6.0)	2 (4.0)
**Erosions**	383	17 (4.4)	73 (19.1)	242 (63.2)	18 (6.3)	17 (4.4)
**Bulges**	57	2 (3.5)	14 (24.6)	16 (28.1)	20 (35.1)	3 (5.3)
**Strictures**	44	1 (2.3)	13 (29.5)	23 (52.3)	4 (9.1)	0 (0)
**Atrophy**	124	1 (0.8)	22 (17.7)	35 (28.2)	3 (2.4)	54 (43.5)
**Diverticula**	23	6 (26.1)	5 (21.7)	8 (34.8)	4 (17.4)	0 (0)

Table S5. Frequency of lesions identified by clinical indication across AI-assisted reading (TOP100)

Lesion	Overall, *N*	TOP100 *N (%)*				
		Overt Bleeding	IDA	IBD	Polyposis/ SB masses	Coeliac
**Angioectasia**	120	17 (14.2)	62 (51.7)	23 (19.2)	1 (0.8)	11 (9.2)
**Ulcer**	231	6 (2.6)	38 (16.5)	173 (74.9)	7 (3.0)	3 (1.3)
**Tumour/Polyp**	25	2 (8.0)	5 (20.0)	2 (8.0)	14 (56.0)	0 (0)
**Active Bleeding**	45	10 (22.2)	20 (44.4)	8 (17.8)	1 (2.2)	4 (8.9)
**Erosions**	274	11 (4.0)	36 (13.1)	197 (71.9)	10 (3.6)	12 (4.4)
**Bulges**	10	2 (20.0)	3 (30.0)	1 (10.0)	3 (30.0)	0 (0)
**Strictures**	24	0 (0)	6 (25.0)	16 (66.7)	1 (4.2)	0 (0)
**Atrophy**	69	0 (0)	9 (13.0)	15 (21.7)	3 (4.3)	35 (50.7)
**Diverticula**	4	1 (25.0)	0 (0)	3 (75.0)	0 (0)	0 (0)
